# The Role of Two-Step Blending in the Properties of Starch/Chitin/Polylactic Acid Biodegradable Composites for Biomedical Applications

**DOI:** 10.3390/polym12030592

**Published:** 2020-03-05

**Authors:** Niyi Gideon Olaiya, Arif Nuryawan, Peter Kayode Oke, H. P. S. Abdul Khalil, Samsul Rizal, P. B. Mogaji, E. R. Sadiku, S. R. Suprakas, Peter Kayode Farayibi, Vincent Ojijo, M. T. Paridah

**Affiliations:** 1 Department of Industrial and Production Engineering, Federal University of Technology Akure, P.M.B. 740, Akure 340282, Nigeria; pkoke@futa.edu.ng (P.K.O.); pbmogaji@futa.edu.ng (P.B.M.); pkfarayibi@futa.edu.ng (P.K.F.); 2 School of Industrial Technology, University Sains Malaysia, Penang 11800, Malaysia; 3 Department of Forest Products Technology, Faculty of Forestry, Universitas Sumatera Utara, Medan 20155, Indonesia; arif5@usu.ac.id; 4 Department of Mechanical Engineering, Universitas Syiah Kuala, Banda Aceh 23111, Indonesia; samsu_r@yahoo.com; 5 Department of Chemical, Metallurgical and Materials Engineering, Tshwane University of Technology, P.M.B. X680, Pretoria 0183, South Africa; ersadiku@gmail.com; 6 DST-/CSIR National Centre for Nanostructured Materials, Council for Scientific and Industrial Research, Pretoria 0001, South Africa; rsuprakas@csir.co.za (S.R.S.); vojijo@csir.co.za (V.O.); 7 Department of Applied Chemistry, University of Johannesburg, Doornfontein, Johannesburg 2028, South Africa; 8 Institute of Tropical Forestry and Forest Products (INTROP), University Putra Malaysia, Seri Kembangan 43400, Malaysia

**Keywords:** two-step mixing, polylactic acid, blend, extrusion, characterization, biodegradable, biomedical

## Abstract

The current research trend for excellent miscibility in polymer mixing is the use of plasticizers. The use of most plasticizers usually has some negative effects on the mechanical properties of the resulting composite and can sometimes make it toxic, which makes such polymers unsuitable for biomedical applications. This research focuses on the improvement of the miscibility of polymer composites using two-step mixing with a rheomixer and a mix extruder. Polylactic acid (PLA), chitin, and starch were produced after two-step mixing, using a compression molding method with decreasing composition variation (between 8% to 2%) of chitin and increasing starch content. A dynamic mechanical analysis (DMA) was used to study the mechanical behavior of the composite at various temperatures. The tensile strength, yield, elastic modulus, impact, morphology, and compatibility properties were also studied. The DMA results showed a glass transition temperature range of 50 °C to 100 °C for all samples, with a distinct peak value for the loss modulus and factor. The single distinct peak value meant the polymer blend was compatible. The storage and loss modulus increased with an increase in blending, while the loss factor decreased, indicating excellent compatibility and miscibility of the composite components. The mechanical properties of the samples improved compared to neat PLA. Small voids and immiscibility were noticed in the scanning electron microscopy images, and this was corroborated by X-ray diffraction graphs that showed an improvement in the crystalline nature of PLA with starch. Bioabsorption and toxicity tests showed compatibility with the rat system, which is similar to the human system.

## 1. Introduction

Biomaterials are used in tissue repair, and the need for these materials is increasing globally. Tissue replacement is commonly used in orthopedics for fractures, including femur, humerus, and bone socket fractures [1,2,3]. These scaffolds have been produced using a combination of both synthetic and natural polymers, such as polylactic acid (PLA), polylactic coglycolide (PLGA), polyvinyl alcohol (PVA), and acrylonitrile-butadiene-styrene (ABS) [4,5,6]. The combination of synthetic and natural polymers has been proven to not be fully biodegradable or biocompatible implant material in repairing bone fractures [7]. Most scaffolds produced from synthetic polymers cause inflammation of the fractured bone as the bone grows [8]. Hence, there is a need for a scaffold that is bioabsorbable by the human body.

The use of a natural polymer as an implant material has various challenges, of which mechanical and degradation properties are of noticeable concern [9]. To resolve these issues, the mechanical and degradation properties of potential natural polymers as a mix in the form of a composite in the place of a synthetic are necessary. In addition, the effect of the blend ratio of new materials and the rate of degradation needs to be established.

Polylactic acid is a polyester, biodegradable polymer from a starch source. It is one of the most consumed natural polymers in industrial applications. It is sourced from starch through a direct condensation process and has been used for several industrial manufacturing products, including packaging and biomedical implants. Polylactic acid has been reported to form a composite that enables the natural growth of cells in bone osteogenic differentiation [10]. Problems with PLA’s poor mechanical properties has limited its use as a scaffold for tissue repair to replace synthetic ones. Polylactic acid, which is prominent among natural polymers for its biodegradability and compatibility, needs to be reinforced with desirable mechanical properties [11].

The literature shows that much work has been done on PLA blending and reinforcement for improved mechanical properties [5,12]. The reinforcement of PLA with ceramics and aluminum improves its mechanical strength remarkably, but biodegradability is reduced, and inflammation is caused when it is used for scaffolding [13]. The blending of PLA with a natural biopolymer has been proposed as a means of improving its mechanical properties [14,15].

Chitin and starch are abundantly available for use [16,17]. They are natural polymers with excellent biodegradable properties and have been used as implants [17,18,19]. Chitin is hydrophobic, while starch, a polysaccharide with a hydrophilic nature, is biocompatible and an absorbable natural polymer. Starch is cheap and readily available. Starch matrix composites have exhibited biocompatibility and biodegradability in medicinal drug release [20]. Chitosan has been reported to neutralize the acidic effect created by the degradation of PLA and improve the ability of PLA to retain mechanical strength during degradation [14]. Additionally, a PLA blend with starch has been reported to improve its biodegradability and compatibility, but with accompanying low mechanical strength as the percentage of starch increases [10]. A comparative analysis of the ternary blend showed that the use of chitosan can improve the adhesion between polylactic acid and starch [21].

On the basis of the reviewed literature, this study sought to find the combined effect of a chitin–starch blend on the mechanical and bioabsorption properties of PLA for biomedical applications. The combined use of PLA, chitin, and starch has not been reported in the literature (hence the novelty of the work).

## 2. Materials and Methods

### 2.1. Materials

The materials were starch, chitin from crab bone, and polylactic acid. Heat-extrusion-grade 4032D PLA from NatureWorks (Minnetonka, MN, USA) was used with a specific gravity of 1.24; a melt flow rate (MFR), in g/10 min (210 °C, 2.16 kg), of 7; and a melt density (g/cc) of 1.08 at 230 °C. The melting temperature, tensile strength, modulus, yield strength, and elongation were 160 °C, 53.5 MPa, 3500 MPa, 60 MPa, and 6%, respectively. Practical-grade chitin (C) (particle size range of 0.50 mm to 0.01 mm) and corn starch (S) (particle size range of 0.50 mm to 0.01 mm) were supplied from Sigma Aldrich (Modderfontein, South Africa). Rats (*Rattus norvegicus*: Wistar rats) were purchased from the Federal University of Technology, Akure, Nigeria.

### 2.2. Composite Processing

The PLA pellets were ground into powder (particle size range of 0.71 mm to 0.045 mm) form using an AA-150 power granulator (Pulian, Taichung, Taiwan) and dried at 60 °C for 4 h with a dry air generator, the Luxor 50 (Motan, Überlingen, Germany). Chitin and starch of varying compositions in powdered form were mixed with a constant percentage of PLA variation using a Thermo Electronic Rheomixer 03 (Thermo Fisher Scientific, Waltham, NJ, USA). The mixture was further mixed and extruded with a parallel c-rotating extruder Process 11 twin-screw extruder (Thermo Fisher Scientific, Waltham, NJ, USA) at a temperature profile of 120 °C, 150 °C, 170 °C, 180 °C, 190 °C, 190 °C, and 200 °C and then quenched in water at room temperature. A feeding rate of 30 sets, a pressure of 35 bar, and a 183 °C melt zone were used for the extruding process. The composite sample filament was pelletized with a Thermo Scientific pelletizer (Thermo Fisher Scientific, Waltham, NJ, USA) and pressed into characterization test shape using a Carver press compression molding machine (Carver, Wabash, IN, USA) at 170 °C for 15 min at a pressure of 100 bar. The test samples were cooled in atmospheric air and stored in Ziploc bags. The composition variation in the current study was based on previous research. In their study, Nasrin et al. [22] reported a uniform mix of PLA blend between 1% and 10% chitin. Additionally, Zakaria et al. [23] have reported that the best mechanical properties of a PLA/chitin composite are with 5% chitin. Five samples were produced (five repeats per sample) with a constant polylactic acid percentage of 92% and chitin (C)/starch (S) ratio variations of 8:0, 6:2, 4:4, and 2:6 (labeled A2, A3, A4, and A5, respectively). Sample A1 had 100% PLA.

### 2.3. Characterization

#### 2.3.1. Tensile Test

An Instron universal testing machine, model 5966 (Instron, Norwood, MA, USA), was used to test the tensile samples prepared at standard dimensions using a Carver press compression molding machine at 170 °C. The test standard used was ASTM D3039-14 (2014) [24] for polymer composites. The grip on the test samples was specified, and the samples were then pulled apart until they broke. The tensile strength, modulus, and yield strength were measured.

#### 2.3.2. Impact Test

A Ceast Resil Impactor 7181 (Corporate Consulting, Service and Instruments (CCSi), Akron, OH, USA) was used for the test per standard ASTM D256-04 (2004) [25]. The impact tester used a hammer on the center of the samples: the energy absorbed, and the difference in the potential energy of the hammer was documented in joules (J). The impact strength of the samples was calculated in joules per square meter (J/m^2^). A standard rectangularly shaped sample was used according to the standard.

#### 2.3.3. Dynamic Mechanical Analysis

A PerkinElmer dynamic mechanical analyzer (DMA) 8000 (PerkinElmer Inc., Ohio, OH, USA) was used to analyze the mechanical behavior of the composite materials. The storage modulus (E’), loss modulus (E’’), and loss factor (Tan δ) were measured. A standard test method, ASTM D4065-06 (2006) [26], was used to obtain results at 1 Hz and 10 Hz to analyze the desired frequency response of the material for tissue repair. The temperature range for the dynamic mechanical analysis was between −50 °C and 150 °C, with a strain factor of 0.3704 and a ramp rate of 5 °C/min, which is standard for polymeric materials.

#### 2.3.4. Microstructural Analysis

A field-emission scanning electron microscope (FE-SEM) JSM7500F (JEOL, Boston, MA, USA) was used to observe the tensile fractured surface morphological properties of the polymer composite. The samples were coated with carbon to improve their conductivity for a visual image in the field-emission scanning electron microscope (SEM).

Additionally, X-ray diffraction (XRD) analysis was performed to obtain the degree of crystallinity of the composite using PANalytical X’Pert PRO X-ray Diffraction (XRD). The X-ray Cu (K-alpha) radiation source had a wavelength of 1.540598 for K-alpha 1 and K-alpha 2, a scan range of 5–90, a generating voltage of 45 volts, and a tube current of 40 A.

#### 2.3.5. Bioabsorption and Biotoxicity Test

Ethical permission was approved by the ethical committee of the Federal University of Technology, Akure, through ethical number FUTA/ETH/007. Standard ethical procedures were adhered to in caring out the biotoxicity test. Female rats were used for the in vivo absorption test. The rats were marked for identification and put in different cages for 5 days before dosing to acclimate them to the environment. After this time, the samples were fed to rats for 14 days, and they were observed at 30-min interval for the first 4 h after every dosage to determine if the composite was bioabsorbable or fatal. The animals were weighed at the start of the experiment, and after 3, 6, 9, and 14 days.

Of the group of five, two rats that were fed samples A3 (6% chitin) and A5 (6% starch), which contained high contents of chitin and starch, were selected for the test, and a third control rat was fed with standard feed. The rats were anesthetized to collect blood samples. After 14 days of extract administration, the animals were euthanized. Chloroform anesthesia was administered to the animals, and blood samples were collected into ethylenediaminetetraacetic acid (EDTA) bottles through cardiac puncture. The blood samples collected were tested for their red blood cell counts (RBCs), white blood cell counts (WBCs), hemoglobin (Hb) concentration, erythrocyte sedimentation rate (ESR), and packed cell volume (PCV) using the following materials: blood samples, microscope, glass slides, a Giemsa stain, capillary tubes, a spectrophotometer, a Leishman stain, EDTA bottles, and a hematocrit centrifuge.

A histological examination was completed on the liver using the method of Palipoch and Punsawad [27]. The liver was preserved for 24 h in buffered formalin (10%) with a neutral pH. It was then washed with ethanol and placed in a metal casket. The casket was stirred with a magnetic stirrer with an alcohol series (70% to 100%). An embedding machine (IHCworld, Woodstock, NY, USA) was used to prepare the liver in paraffin. The blocks of paraffin formed were sectioned using a rotary ultramicrotome and distributed on the glass slide. The glass slide was dried overnight and stained with hematoxylin and eosin. The slide was observed under a light microscope.

## 3. Results

### 3.1. Tensile Properties

**Figure 1:**
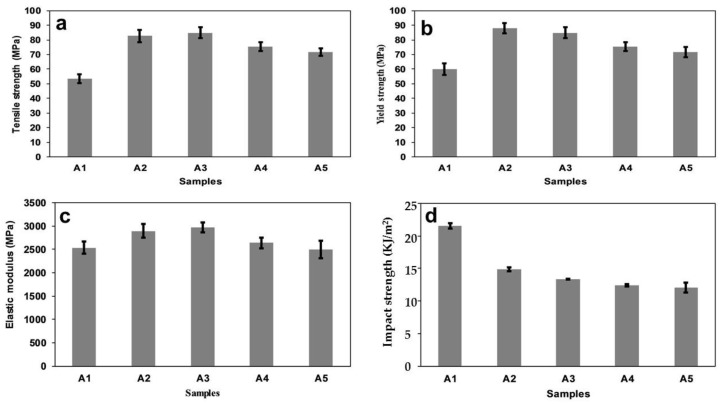
Mechanical properties: (**a**) tensile strength, (**b**) yield strength, (**c**) elastic modulus, and (**d**) impact strength of samples A1 (neat PLA), A2 (PLA/8% chitin), A3 (PLA/6% chitin/2% starch), A4 (PLA/4% chitin/4% starch), and A5 (PLA/2% chitin/6% starch).

Graphs of the tensile strength, yield strength, and elastic modulus are shown in Figure 1a–c, respectively. Generally, there was a significant improvement in the tensile and yield strengths of the composite compared to neat polylactic acid (A1), but there was a drop from that to PLA/8% chitin (A2). The tensile and yield strengths improved with a decrease in starch content (A2 to A5). Additionally, the elastic modulus decreased with starch content, although sample A4 (PLA/4% C/4% S) (C is chitin and S is starch) showed a more significant drop. The neat PLA used had a tensile strength of 53.5 MPa, and the blend’s tensile strength increased to 84.94 MPa. When compared to PLA/8% chitin (A2), an increase in tensile strength was first noticed in A3 (PLA/6% C/2% S), but there was then a decrease to A5 (PLA/2% C/6% S) with an increase in starch percentage. A similar trend was observed with the yield strength. The neat PLA had a yield strength of 60 MPa, which showed a significant percentage increase compared to the peak of A3 (PLA/6% C/2% S) (Figure 1b). The increase in the strength of the blend could be linked to the compatibility and miscibility of the polymer blend, which was confirmed by the DMA results. The same increasing trend in the tensile and yield strength values has been reported in the literature [28,29]. The tensile strength of the PLA/chitin/starch (A3 to A5) was in the range of material used for the tissue repair of cancellous/trabecular bone [30].

The value of the elastic modulus is essential to the mechanical strength of the materials used for bone tissue repair [30]. Compared to the neat PLA, all samples showed a significant improvement in terms of the elastic modulus (Figure 1c). The elastic modulus of the samples decreased with an increase in the percentage of starch (decreasing chitin), with a peak value at A3 (PLA/6% C/2% S). The elastic modulus value showed that the stiffness of the PLA was enhanced with the addition of chitin and reduced starch content. The stiffness of the composite could be attributed to the combined effect of the brittleness of starch and the enhanced toughness of chitin [22,31]. The values of the elastic modulus corroborated the possible use of the material for the repair of cancellous bone. Cancellous (trabecular) bone is the type of bone in the human body that is most prone to fracture [32].

Generally, the impact strength of neat PLA has been reported to increase with chitin and reduce with starch content above 10% due to incompatibility with PLA [33]. Minimal work has been reported on the impact strength from a blend of 10% chitin/starch with PLA. In this study, the impact strength compared to neat PLA was observed to decrease with the addition of starch and a reduction of chitin.

### 3.2. Dynamic Mechanical Analysis

**Figure 2:**
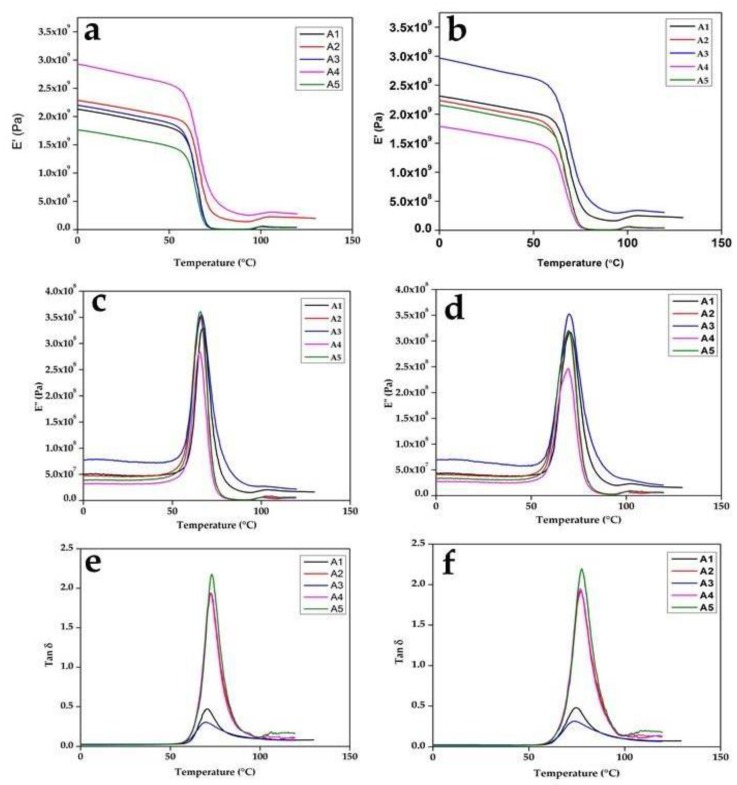
Dynamic mechanical analysis (DMA) at 1 Hz for (**a**) E’, (**c**) E”, (**e**) and tan δ; and at 10 Hz for (**b**) E’, (**d**) E”, (**f**) and tan δ for samples A1, A2, A3, A4, and A5.

The results of the dynamic analysis at 1 Hz and 10 Hz are shown in Figure 2. The dynamic mechanical analysis of the composite at 1 Hz is shown in Figure 2a,c,e, while the DMA at 10 Hz is shown in Figure 2b,d,f. The composite blend showed similar behavior at both frequencies. Generally, the dynamic modulus curve showed an increase in the storage modulus (E’) value below the glass temperature (Tg) region and a lower value in the rubbery plateau (Figure 2a,b). The neat PLA storage modulus curve is shown between the samples. Samples A2 (PLA/8% C), A3 (PLA/6% C/2% S), and A4 (PLA/4% C/4% S) had values above neat PLA (A1), which is an indication of an improvement in the elastic properties of PLA. Sample A5 (PLA/2% C/6% S) exhibited a curve below neat PLA, which showed a tendency toward a viscous nature.

A drastic drop in the storage modulus of samples is characteristic of an amorphous substance. The value of the storage modulus was generally higher than the loss modulus, which was an indication that the elastic nature of the polymer dominated the viscous nature. A drastic drop in the transition region corroborated the amorphous nature of the composite blend. Additionally, the curves had a single transition that showed the miscibility of the blended polymers. The miscibility of the blend could also be seen in the loss modulus and loss factor graph, which has a single peak and represents the glass transition temperature. The strength of the intermolecular forces of the molecules determined the value of the modulus in the glassy state. The value of the modulus was high, which showed that there was high strength and miscibility between the PLA, chitin, and starch. The graph of E” and the loss factor (tan ∂), which has a single peak for each of the composites, shows the characteristics of the glass transition temperature. The value of the midpoint of the peak in each of the loss factor curves (Figure 2e,f) is the glass temperature. When the Tg was higher, the material was more resistant to heat.

The value of the loss factor was low compared to neat PLA, which was an indication of improved interfacial bonding (Figure 2e,f). The lower values of the loss factor were more pronounced with samples A2 (PLA/8% C) and A4 (PLA/4% C/4% S) and less pronounced with A3 (PLA/6% C/2% S) because the storage modulus was high. The high tan δ value of sample A1 (100% PLA) was largely due to its high loss modulus value (Figure 2c), while that of sample A5 was due to its low storage modulus value (Figure 2a,b). The interfacial adhesion of a polymer mix has a directly proportional relationship with its damping properties. Here, interface adhesion reduced with starch percentage, except in sample A4 (PLA/4% C/4% S) (due to the value of its storage modulus). The storage modulus value of sample A4 (PLA/4% C/4% S) may have been due to an equal percentage of chitin and starch in the blend. The tan δ value of the samples was much lower with chitin, which showed interfacial adhesion, and this improved with an increase in the percentage composition of chitin in the composite. Generally, the tan δ graph was characterized by a broader width (Figure 2e,f). This behavior suggested that there was molecular relaxation (even stress distribution) in the composite [34]. The broader tan δ relaxation in the case of the samples containing more starch was actually the effect of the overlapping relaxation of PLA and starch. Moreover, the increased tan δ of the samples containing higher amounts of starch was actually the effect of microscopic adhesion failure in the interface between PLA and starch (as opposed to any molecular movements). Generally, the movement of molecules at the interfacial region and that of the constituents affect the damping behavior of a material [35]. Additionally, the value of tan δ indicated the viscous property of the polymer. Samples A1 (100% PLA) and A5 (PLA/2% C/6% S) tended toward a viscous liquid, while samples A2 (PLA/8% C), A3 (PLA/6% C/2% S), and A4 (PLA/4% C/4% S) tended toward an elastic solid. A viscous liquid dissipates energy, while an elastic solid stores energy.

### 3.3. Morphological Properties

**Figure 3:**
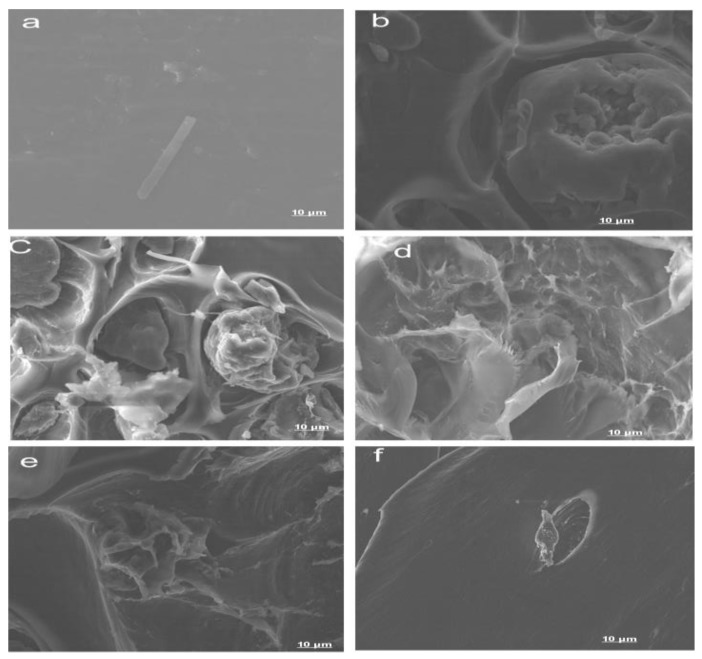
Morphological properties of (**a**) A1 (neat PLA surface), (**b**) A2 (PLA/8% chitin), (**c**) A3 (PLA/6% chitin/2% starch, (**d**) A4 (PLA/4% chitin/4% starch), (**e**) A5 (PLA/2% chitin/6% starch), and (**f**) a neat PLA-fractured surface.

The freeze-fractured SEM surface morphologies of all blends are presented in Figure 3. The neat PLA (sample A1 in Figure 3a) showed a smooth, compact surface. Figure 3b–e corresponds to samples A2 (PLA/8% C), A3 (PLA/6% C/2% S), A4 (PLA/4% C/4% S), and A5 (PLA/2% C/6% S).

**Figure 4:**
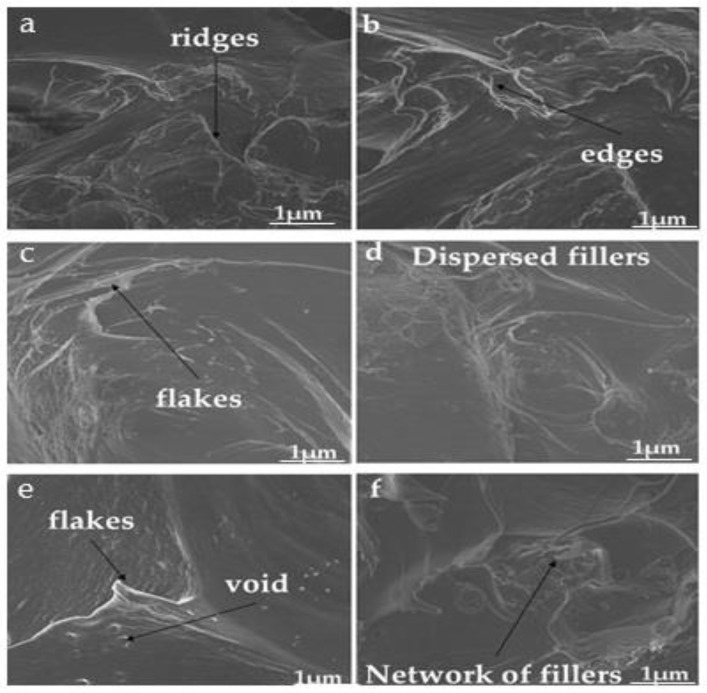
Morphological properties of samples showing edges, small cleavages, tear ridges, and a network of dispersion (the blends with PLA).

The SEM images showed that blending chitin and starch with PLA (Figure 3b through Figure 3e) produced a dispersed morphology, where the starch and chitin were dispersed in the PLA. The SEM images of the blend (Figure 3a–e) were characterized with edges, small cleavages, tear ridges, and a network of dispersion (the blends with PLA) (Figure 4) [36]. The images also showed the high dispersion and miscibility of the three polymers, with small voids and patches of blending. The high miscibility revealed by the DMA test is shown in the SEM images. The images revealed high interfacial bonding and agglomerated particles at low magnification. The images reflected an accurate mix and compatibility of the matrix with the reinforcement, which accounted for the significant increase in the mechanical properties of the composite compared to the neat PLA matrix [37,38]. The SEM images showed a uniform flaky distribution of the blended materials, with negligible voids.

### 3.4. XRD Analysis

**Figure 5:**
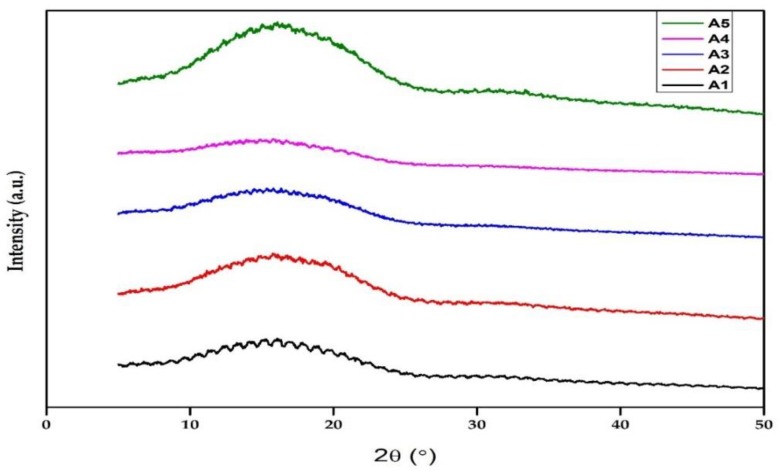
XRD of the composites: (**A1**) (neat PLA), (**A2**) (PLA/8% chitin), (**A3**) (PLA/6% chitin/2% starch), (**A4**) (PLA/4% chitin/4% starch), and (**A5**) (PLA/2% chitin/6% starch).

The degree of crystallinity and an X-ray diffraction plot for all samples are shown in Table 1 and Figure 5. The diffractive peaks of the blending showed a slight change in position compared to neat PLA. The shift indicated that the addition of chitin and starch particles exerted a considerable effect on the crystalline properties of PLA. An increase in the starch or chitin blend caused a slight noticeable improvement in the crystallinity of the neat PLA [22,39]. A small improvement was noticed with an increase in starch content, as shown in the degree of crystallinity (DOC) of sample A5 (PLA/2% C/6% S). A slight increase in the crystallinity of PLA has also been reported by Lv et al. [39] and Ayana et al. [40] (this was thought to be due to the nucleation effect of starch). The increase in crystallinity resulted in a more thermal stable composite, as seen in the DMA analysis. In addition, the reduction in the percentage of chitin seemed to be nullified by the addition of starch, as the DOC increased. This shows that the contribution of starch to the improved crystalline properties of PLA was more significant than that of chitin.

### 3.5. Biotoxicity Properties

**Figure 6:**
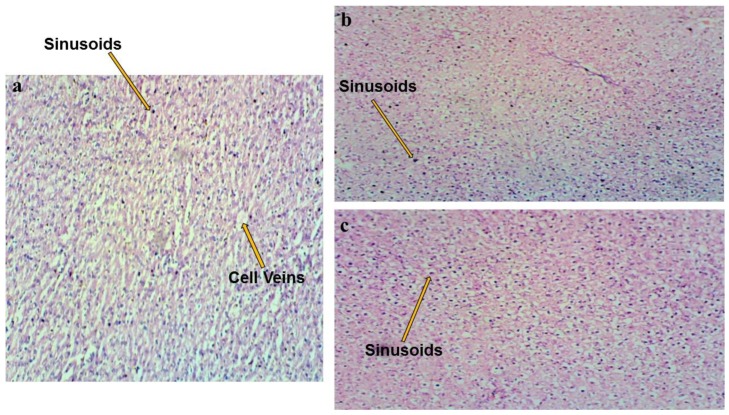
Liver light microscopy of a histological examination (hematoxylin and eosin stain) of the rats: (**a**) control (standard feed), (**b**) green (PLA/6% chitin/2% starch), and (**c**) red (PLA/2% chitin/6% starch).

The results of the absorption of the composite samples (A1 to A5) are shown in Table 2. The table records the percentage increase in the weight of the rats at intervals from 1 day to 14 days. Generally, each of the rats increased in weight with all of the samples. The control rat sample had the highest percentage increase in weight, followed by the rat fed with sample A2 (PLA/8% C). The weight of the rats increased with the percentage of chitin and reduced with an increase in starch content. Additionally, there was no reduction in the weight of the rats throughout the experiment. Physical observations of the rats’ faces showed no visible traces of the composite pellets. No mortality or weakness was recorded during the absorption test. The results of the acute toxicity test using a hematology (full blood count) test and a liver histology architecture of blood from select rats fed with samples A3 and A5 (based on a high content of chitin and starch) are shown in Table 3 and Figure 6, respectively. The hematology test showed that white blood cells did not significantly increase in the sample rats fed with A3 (PLA/6% C/2% S) and A5 (PLA/2% C/6% S).

A significant increase in the white blood cell count may mean the presence of a parasite or toxin [37]. However, the control samples had a healthier blood count than did the other rats, which was probably the result of proper feeding [41].

A change in the liver architecture under a light microscope is usually ascribed to tension in the liver caused by a parasite or toxic substance [37]. Liver architecture images under a light microscope for the control rats and the rats fed with a high chitin and starch content are shown in Figure 6a–c. Healthy central veins and sinusoids characterized the control rats. The architecture image of the rats fed with sample A3 (PLA/6% C/2% S) (high chitin content) showed no significant change in liver architecture. Additionally, Figure 6c, which is of the rat fed with sample A5 (PLA/2% C/6% S) (high starch content), showed no change in terms of typical liver architecture [42]. These characteristics were similar to the control rats. The typical architecture of hepatocytes with well-organized sinusoids and a central vein were noticed in the control rats, while the other rats showed well-organized sinusoids.

## 4. Conclusions

1. Starch/chitin/PLA composites were successfully developed using a two-step pre-mix;
2. The mechanical properties of the polymer composites increased significantly more than did those of the neat PLA. The mechanical properties of the composites were improved using this method (with varying starch/chitin/PLA percentages). Sample A3 (PLA/6% C/2% S) had optimal strength and a suitable combination of the blend. This material can be used for trabecular tissue repair or any tissue repair (below the cut-off values of the tensile strength and modulus);
3. The brittleness of neat PLA was reduced with the addition of the blend in all of the samples;
4. The DMA of the composites showed a good storage modulus for stiffness, reduced loss factor, and good miscibility. It also showed a single peak, which showed the compatibility of the polymer blend. The glass temperature from the loss factor peak showed that the material was not suitable for high-temperature biomedical applications;
5. The composite, which was molded using a Carver press, showed a uniform distribution of the natural polymer blend in its physical appearance. The SEM images showed a network distribution of the chitin/starch blend in PLA. Though there were small voids, the images showed a homogenous blend of chitin/starch with PLA;
6. The amorphous characteristics of neat PLA caused no significant change in the blend; and
7. The material was absorbable and nontoxic according to the results of the acute toxicity test. Thus, full clinical analyses are recommended.

## Figures and Tables

**Table 1:** Degree of crystallinity (DOC) of the composites.

Samples	DOC (%)
A_1_	7.18
A_2_	7.10
A_3_	7.55
A_4_	8.56
A_5_	8.42

**Table 2:** Percentage weight increases of the rats.

Rat Color Identification	Sample	Weight Increase (%)	Weight Increase (%)	Weight Increase (%)	Weight Increase (%)
		3 Days	6 Days	9 Days	14 Days
Blue rat	A_2_	26	38	90	94
Green rat	A_3_	7	19	20	111
Black rat	A_4_	23	52	81	99
Red rat	A_5_	12	24	64	67
Control	Feed	35	74	95	134

**Table 3:** Complete blood cell counts.

Rat	PCV	Hb	WBC	RBC	MCV	MCH	MCHC	Differential Count				
	(%)	(g/L)	(10^9^/L)	(10^12^ g/L)	(fL)	(pg)	(g/dL)	N	L	E	M	B
Control	48	16.1	6.8	4.57	105.03	35.54	33.54	50	48	1	1	0
Green	34	11.4	5.9	3.37	100.89	33.82	38	35	62	2	1	0
Red	30	10.2	5.4	3.32	90.36	3.4	34	50	48	1	1	0

N: neutrophils, L: lymphocytes, E: eosinophils, M: monocytes, B: basophils; red blood cell count (RBC), white blood cell count (WBC), hemoglobin (Hb) concentration, erythrocyte sedimentation rate (ESR), packed cell volume (PCV), mean corpuscular volume (MCV), mean corpuscular hemoglobin (MCH), mean corpuscular hemoglobin concentration (MCHC).
